# Scalar position characteristics and translocation rates of different lateral wall electrodes within the cochlear basal turn: a cone beam computed tomography study

**DOI:** 10.1007/s00405-026-10431-5

**Published:** 2026-08-07

**Authors:** Irumee Pai, Paolo Rondi, Isobel Morley, Emily Hocknell, Andrew Hoey, Sebastien Ourselin, Christos Bergeles, Stephen Connor, Rithvik Reddy

**Affiliations:** 1 St. Thomas’ Hearing Implant Centre, Guy’s and St. Thomas’ NHS Foundation Trust, 2nd Floor Lambeth Wing, London, UK; 2 School of Biomedical Engineering and Imaging Sciences, King’s College London, London, UK; 3 Department of Radiology, Radiological Sciences and Public Health, University of Brescia, Brescia, Italy; 4 Department of Radiology, Guy’s and St. Thomas’ NHS Foundation Trust, London, UK

**Keywords:** Lateral wall cochlear implant electrodes, Scalar translocation, Cone-beam computed tomography, Basal turn cochlear anatomy, Angular insertion depth

## Abstract

**Purpose:**

This retrospective study systematically compared scalar position characteristics and translocation (STL) rates within the cochlear basal turn (CBT) across three different lateral wall (LW) electrodes.

**Methods:**

The study included 148 morphologically normal cochleae implanted with Advanced Bionics SlimJ (ABSJ, *n* = 51), Cochlear Slim Straight (CSS, *n* = 51), or MED-EL FLEX (MFX, *n* = 46). Cochlear dimensions (distances A and B) and angular insertion depth (AID) were measured by two observers. Scalar position was assessed on postoperative cone-beam CT at 90°, 180°, 270°, and 360° by three observers using a five-point scale (1–2 = scala vestibuli (SV), 3 = central, 4–5 = scala tympani (ST)).

**Results:**

Distances A and B were comparable across groups (*p* > .05). Mean AID was significantly greater for MFX (*p* < .001). MFX had higher median scalar scores than CSS at every angle (all Bonferroni-adjusted *p* < .001) and than ABSJ at 180°, 270°, and 360° (adjusted *p* < .001). ST retention (all four median scores ≥ 3) was observed in 92.2% of ABSJ, 78.4% of CSS, and 100% of MFX cases, while STL (median score decreased to 1–2 and remained ≤ 3) occurred in 7.8%, 15.7%, and 0%, respectively. MFX demonstrated significantly lower STL and higher ST retention rates than CSS (adjusted *p* = .018 and *p* < .003, respectively). No independent associations of distance A, distance B, or AID with either STL or ST retention were identified (all *p* ≥ .54).

**Conclusion:**

Significant differences in scalar position and STL rates were observed among LW electrodes, with MFX showing the most stable ST placement.

**Supplementary Information:**

The online version contains supplementary material available at https://doi.org/10.1007/s00405-026-10431-5.

## Introduction

Cochlear implantation has transformed the management of sensorineural hearing loss in adults and children who do not derive adequate benefit from acoustic hearing aids. The indications for cochlear implantation have been expanding globally, with over one million individuals implanted as of 2022 [1]. As individuals with residual hearing are increasingly considered for implantation, there is growing emphasis on preserving the delicate structures within the cochlea. Intracochlear trauma during electrode insertion not only negatively affects hearing preservation [2–4] but may also result in fibrosis and neo-ossification, leading to compromised cochlear implant (CI) performance and reduced potential for future hearing-regenerative interventions [5–7].

Scalar translocation (STL), defined as the misplacement of the CI electrode from the scala tympani (ST) into the scala vestibuli (SV) or scala media (SM), is associated with damage to the basilar membrane, organ of Corti, and stria vascularis [8–11]. It is also linked to poorer speech perception and loss of residual hearing [12–14]. The marked individual variation in cochlear anatomy influences the positioning of the electrode within the cochlear chambers. The orientation of the round window (RW), size and morphology of the cochlear basal turn, and facial recess anatomy have all been shown to affect the electrode position within the cochlea [15–17]. The surgical approach for electrode insertion, the insertion force, and the insertion velocity have also been reported as factors that affect electrode positioning [18–21].

Definitive confirmation of scalar translocation requires histopathologic examination, which is not feasible in vivo; clinical assessment therefore relies predominantly on postoperative imaging, most commonly using computed tomography (CT) or cone-beam CT (CBCT). A practical method entails a linear grading scale (1–5) to reflect the antero-posterior relationship within the cochlear lumen on oblique parasagittal images, with scalar translocation inferred when two or more electrode contacts occupy the anterior half of the lumen [18, 22]. This approach, however, offers only a simplified representation relative to the histological classification of intracochlear trauma proposed by Eshraghi et al. [23].

The published literature to date suggests that lower rates of STL are observed with lateral wall LW electrodes than with perimodiolar (PM) electrodes [11, 19, 24]. In a recent meta-analysis, Jwair et al. [4] found the STL rate of LW electrodes to be approximately 7%, as compared to 43% with perimodiolar (PM) electrodes. However, there is little published literature that directly compares lateral wall electrodes from different manufacturers in their STL rates.

In the current study, the authors aimed to systematically assess the scalar position characteristics and STL within the cochlear basal turn on postoperative CBCT, and to directly compare LW electrodes from three different manufacturers.

## Materials and methods

### Ethical considerations

This retrospective study underwent local institutional review and was approved, including waived informed consent (Electronic Record Research Interface, IRAS ID: 257283; REC reference: 20/EM/0112; approval date: 26/01/2023). The study was conducted strictly in accordance with the relevant guidelines and regulations.

### Study cohort

The Auditbase, Picture Archiving and Communication System (PACS) Sectra and Electronic Patient Record databases were searched for all adult and paediatric patients implanted with LW electrodes at our centre between December 2013 and December 2024, who had undergone postoperative CBCT. Exclusion criteria were: incomplete clinical data, poor imaging quality, congenital cochlear anomalies, pathologies affecting cochlear lumen patency, electrode insertion via cochleostomy, explantation/re-implantation surgery, buckling or tip fold-over, and partial insertion, defined as the presence of active electrode contacts lateral to the RW on postoperative CBCT.

The study cohort was divided into three groups based on the device manufacturer: Advanced Bionics SlimJ (ABSJ; Advanced Bionics LLC, Valencia, CA, USA), Cochlear Nucleus Slim Straight Electrodes 422/522/622 (CSS; Cochlear Ltd., Sydney, Australia), and MED-EL FLEX24/26/28 (MFX; MED-EL GmbH, Innsbruck, Austria). Data were collected on age at implantation, implanted ear side, operating surgeon, preoperative and postoperative hearing levels, angular insertion depth (AID), and cochlear basal turn dimensions (distance A and distance B) to evaluate whether the three groups were comparable in demographic and anatomical parameters and to identify any potential confounding factors influencing electrode scalar position. Pure-tone audiometry (PTA) was performed according to the British Society of Audiology recommended procedures. The hearing levels were presented as the four-frequency pure-tone average of unaided air conduction thresholds over 0.5, 1, 2 and 4 kHz (PTA4) and as the low-frequency average over 0.25, 0.5 and 1 kHz (LFPTA3). Where no response was obtained at the maximum output of the audiometer at a given frequency, the threshold was assigned as the maximum output plus 5 dB for the purposes of hearing level calculation.

### Measurement of cochlear basal turn dimensions and AID on CBCT

**Fig. 1:**
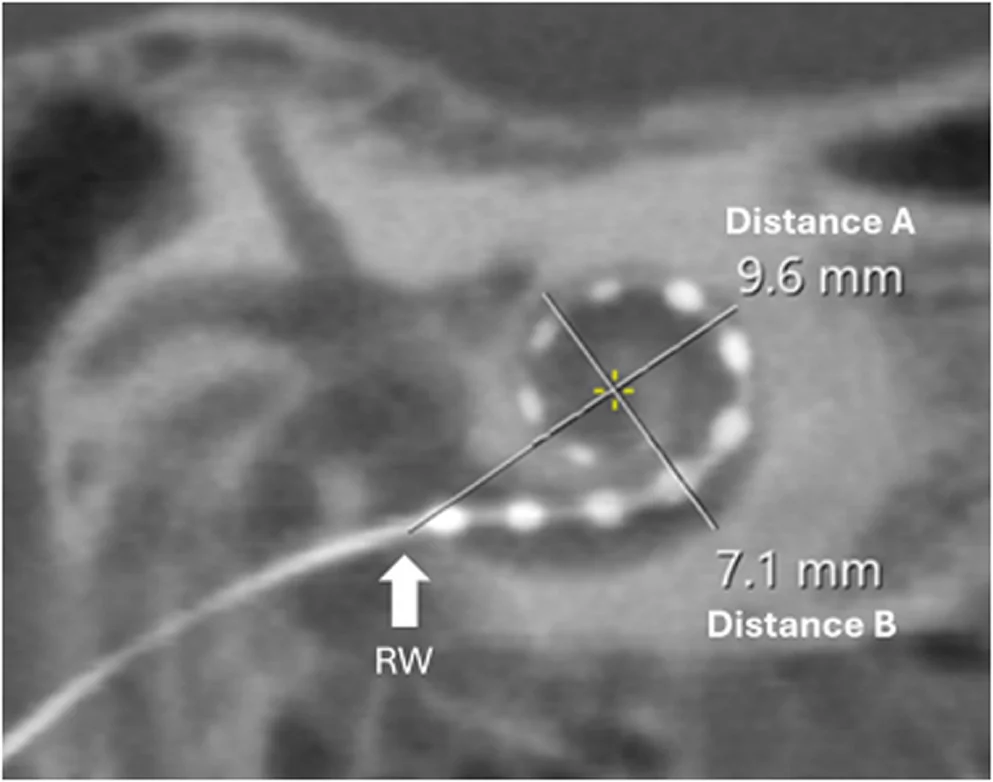
Distance A is measured as the greatest distance from the mid-round window (RW, white arrow) to the opposite wall of the basal turn through the mid-modiolar axis (crosshair). Distance B is measured as the distance perpendicular to distance A through the same mid-modiolar axis

**Fig. 2:**
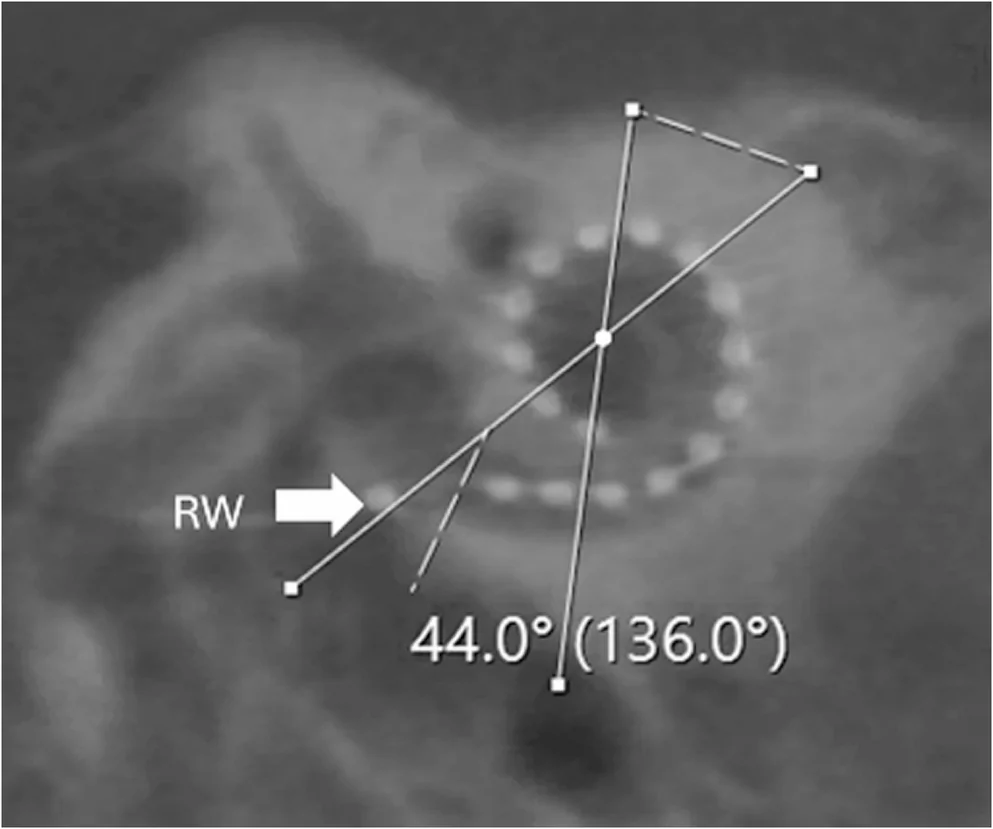
A 2-mm slab thickness average reconstruction is performed with a standardised window to include all electrode contacts within the section thickness. A line is placed in the axis of distance A from the mid-round window (RW, white filled arrow) through the mid-modiolar axis (white dot), which bisects the more distal cochlea at the 360° point. An angle is then measured between this point and the most distal electrode contact

CBCT imaging was performed using a 3D Accuitomo 170 (J. Morita, Kyoto, Japan), model MCT-1, type EX-2F17, with parameters: voltage 80 kV, current 10 mA, and voxel size 0.125 (0.125 × 0.125 × 0.125). The measurement of cochlear basal turn dimensions and AID was performed on the PACS Sectra software, in accordance with established protocols [18, 25]. For the basal turn measurements, distance A was measured as the greatest distance between the RW and the LW through the mid-modiolar axis, and distance B as the perpendicular distance to distance A through the same mid-modiolar axis on a double-oblique paracoronal reformatted image [26] (Fig. 1). Both distances were recorded to the nearest 0.1 mm. The angle measurement tool of the PACS Sectra software was used to measure the AID of the most apical electrode contact to the nearest degree, around the mid-modiolar axis on a double-oblique coronal reformatted image with a 2 mm average slab reconstruction, designed to demonstrate the entire electrode array with the RW as the 0° point. Where the electrode was inserted beyond the basal turn and into the middle turn of the cochlea, the angle between the RW and the most apical electrode contact through the mid-modiolar point was added to 360° (Fig. 2). All measurements were performed independently by two observers, an otologist and a neuroradiologist, and the mean of the two readings was used for the statistical analysis.

### Evaluation of intracochlear position and scalar translocation

**Fig. 3:**
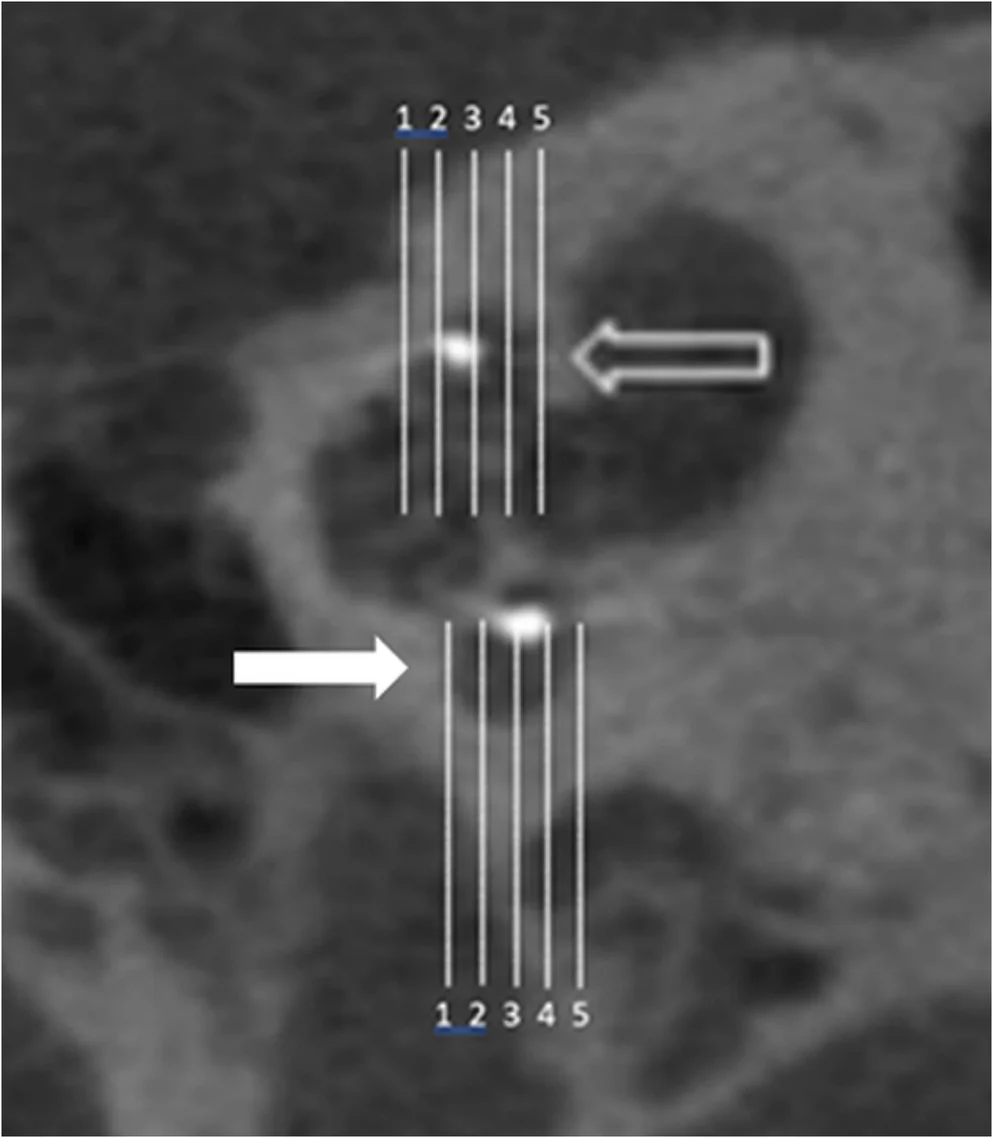
At 90° (white filled arrow), the electrode is located between 25 and 50% of the posterior lumen (score 4). At 270° (white open arrow), the electrode is located between 25 and 50% of the anterior lumen (score 2).

The position of the electrode array within the scala chambers was assessed at four locations within the basal turn of the cochlea, at 90°, 180°, 270° and 360° AID, on an oblique parasagittal reformat parallel to the mid-modiolar axis, reflecting its anterior (scala vestibuli, SV)-to-posterior (ST) relationship within the cochlear lumen. The location of the electrode was scored on a linear scale of 1 (anterior 0–25% of lumen) to 5 (posterior 0–25% of lumen), as summarised in Table 1 and illustrated in Fig. 3. Scores 1 and 2 indicated a position in the SV compartment, scores 4 and 5 a position in the ST compartment. A score of 3 indicated a position within the central lumen. The assessment was confirmed by scrolling through adjacent slices. The scoring was performed independently by three observers, two otologists and a neuroradiologist, and the median of the three scores was used for the statistical analysis.

**Table 1:** Scoring of the scalar location within the cochlear lumen

Score	Location of the electrode
1	Anterior 25% of the lumen
2	Anterior 25–50% of the lumen
3	Central within the lumen
4	Posterior 25–50% of the lumen
5	Posterior 0–25% of the lumen

In a previous publication on the assessment of STL on postoperative CT, Connor et al. [18] defined ST placement of the electrode array as two or more scores of 4 or 5, whilst STL was defined as two or more scores of 1 or 2. Two or more scores of 3 were classified as indeterminate cases. For the current study, the definitions were further refined a priori to better align with the histological classification proposed by Eshraghi et al. [23]. When the scores across all four quadrants were 3 or higher, it was categorised as Class A and interpreted as placement within the ST throughout the basal turn (comparable to Eshraghi grade 0). When the median score decreased to 2 or 1 at a single angle but subsequently increased to 4 or 5, it was categorised as Class B and interpreted as elevation or rupture of the basilar membrane (comparable to Eshraghi grades 1 and 2). When the median score decreased to 2 or 1 and remained ≤ 3 at subsequent angles, it was categorised as Class C and interpreted as translocation (STL) (comparable to Eshraghi grades 3 and 4). This modified classification is summarised in Table 2. If the first decrease to 2 or 1 occurred at 360°, additional scoring was performed beyond 360° up to the most apical electrode contact.

**Table 2:** Comparison of proposed CBCT and histological classifications

CBCT-based classification (current study)	Histological classification (Eshraghi et al. 2003)
Class A: All four quadrant scores are ≥ 3	Grade 0: No observable trauma
Class B: Median score drops to 1 or 2, then rises to 4 or 5	Grade 1: Elevation of basilar membrane Grade 2: Basilar membrane rupture
Class C: Median score drops to 1 or 2, and remains ≤ 3	Grade 3: Electrode displaced into scala vestibuli Grade 4: Fracture of osseous spiral lamina

### Statistical analysis

Data were analysed using IBM SPSS Statistics version 31 (IBM Corp., Armonk, NY, USA). The significance level was set at *α* = 0.05 (two-tailed) for all statistical tests.

Descriptive statistics included means ± standard deviation (SD) for continuous variables (age at implantation, preoperative and postoperative hearing levels (PTA4 and LFPTA3 thresholds), distance A, distance B and AID), and frequencies and percentages for categorical variables (electrode array group, implanted ear side, operating surgeon). The Shapiro-Wilk test showed significant deviation from normality for age at implantation and hearing thresholds in some groups, whereas distance A, distance B and AID were approximately normally distributed. Homogeneity of variances was evaluated using Levene’s test. Comparisons among the three groups were performed using one-way analysis of variance (ANOVA). For variables showing a significant overall ANOVA result, Tukey’s honestly significant difference (HSD) post hoc test was used when homogeneity of variances was satisfied, and Games-Howell post hoc testing was used when the homogeneity assumption was violated. Although age deviated from normality and hearing levels showed variable normality across groups, the relatively large group sizes (*n* > 45 per group) justified the use of parametric ANOVA, which is robust to moderate departures from normality. The association between the implanted ear side and electrode array group was assessed using the Pearson chi-square test.

For the scalar scores at each angular position, the median of the three reader scores was used for analysis. The Kruskal-Wallis test was used to compare the three electrode array groups. When a statistically significant overall difference was detected, post hoc pairwise analyses were performed using Mann-Whitney U tests to identify which groups differed significantly.

Intracochlear position classifications were compared using non-parametric exact methods to accommodate small cell counts and potential zero-frequency categories. Two independent comparisons were performed across the three electrode array groups: one assessing the proportion of cases with scalar translocation (STL vs. no-STL), and another evaluating the proportion of arrays located entirely within the scala tympani (ST vs. not-ST). These parallel analyses were undertaken because intracochlear positions exist along a three-class continuum (Class A = ST retention, B = elevation or rupture of the basilar membrane, and C = STL) and it is not self-evident whether Class B should be regarded as more closely related to complete ST retention or to definite STL. Exact *p*-values were obtained using the Fisher-Freeman-Halton procedure for multi-group comparisons, and when a statistically significant overall difference was identified, post hoc pairwise analyses with two-sided Fisher’s exact tests were conducted to determine which electrode array groups differed significantly. Where relevant, Bonferroni adjustment was applied to control for multiple comparisons. The primary analysis included all cases. In addition, a sensitivity analysis was performed, excluding the cases in which the score decreased to 2 or 1 for the first time at 360°.

Inter-observer reliability between the two readers for the measurements of distance A, distance B and AID was assessed using the intraclass correlation coefficient (ICC) based on a two-way random-effects model with an absolute-agreement definition. For the scalar scores from the three readers, inter-observer reliability was evaluated using both the ICC and pairwise weighted Cohen’s κ values calculated for all reader pairs.

## Results

### Study cohort

The search method initially identified 190 ears. Of these, 42 ears were excluded for the following reasons: active electrode contacts lateral to the RW (*n* = 8/42); explantation/re-implantation surgery (8/42); electrode insertion via cochleostomy (7/42); compromised patency of the cochlear basal turn (7/42; cochlear involvement of tumor in neurofibromatosis type 2, 3; fibrosis, 2; far-advanced otosclerosis, 2); poor imaging quality (6/42); incomplete partition type 2 (4/42); and insufficient clinical data (2/42). The distribution of excluded ears across the three electrode array groups was as follows: ABSJ 27/42; CSS 11/42; and MFX 4/42. Details of the reasons for exclusion by array type are provided in Supplementary Table 1. The final analysis included 148 ears (69 left, 79 right) in 137 patients, all of which were morphologically normal cochleae. The mean age at implantation was 52.9 years ± 23.4 (standard deviation, SD). There were a total of six operating surgeons, with all but three cases (97.9%) having been performed by four of the six surgeons. Of the 148 cases, RW membrane incision was performed for electrode insertion in over 95% (142/148), with drilling of the crista fenestrae being required in the remaining six cases (two per each of the three electrode array groups).

### Demographic and anatomical comparisons

There were 51 ears in the ABSJ group (32 right; 19 left), 51 ears in the CSS group (22 right; 29 left), and 46 ears in the MFX group (25 right; 21 left). The mean age at implantation was 53.9 ± 23.3 years, 46.1 ± 24.9 years, and 59.5 ± 20.1 years, respectively. The audiometric data were available in all 148 cases preoperatively and in 122/148 cases postoperatively (ABSJ 40/51; CSS 42/51; MFX 39/46). The mean preoperative and postoperative (respectively) PTA4 thresholds were as follows: 100 ± 15 and 112 ± 10 dB HL in the ABSJ group; 95 ± 15 and 109 ± 15 dB HL in the CSS group; 95 ± 16 and 109 ± 12 dB HL in the MFX group. The mean preoperative and postoperative (respectively) LFPTA3 thresholds were as follows: 88 ± 18 and 102 ± 16 dB HL in the ABSJ group; 78 ± 22 and 96 ± 20 dB HL in the CSS group; 74 ± 27 and 93 ± 20 dB HL in the MFX group. Mean distance A was 9.1 ± 0.4 mm in the ABSJ group, 9.1 ± 0.5 mm in the CSS group, and 9.2 ± 0.4 mm in the MFX group. The mean distance B was 6.7 ± 0.4 mm, 6.9 ± 0.4 mm, and 6.8 ± 0.4 mm, respectively. Mean AID was 436 ± 46°, 426 ± 53°, and 507 ± 58°, respectively.

There was no difference among the three electrode array groups in implanted ear side (χ² = 3.97, df = 2, *p* =.138), distance A (F(2, 145) = 0.093, *p* =.911), or distance B (F(2, 145) = 1.650, *p* =.196). There was also no difference in preoperative (F(2, 145) = 2.094, *p* =.127) or postoperative (F(2, 118) = 0.687, *p* =.505) PTA4 thresholds. Formal statistical analysis of surgeon-specific scalar outcomes was not possible due to insufficient event counts per surgeon for either STL vs. no-STL or ST vs. not-ST analysis.

The one‑way ANOVA preoperative LFPTA3 thresholds showed a significant difference among the three electrode array groups (F(2, 145) = 5.295, *p* =.006). Games-Howell post hoc testing demonstrated significantly higher preoperative LFPTA3 thresholds in the ABSJ group compared with the CSS group (*p* =.025) and the MFX group (*p* =.010), whereas LFPTA3 thresholds did not differ significantly between the latter two electrode array groups (*p* =.758). There was no difference in postoperative LFPTA3 thresholds among the three groups (F(2, 118) = 2.365, *p* =.098).

The one‑way ANOVA for age at implantation showed a significant difference among the three electrode array groups (F(2, 145) = 4.19, *p* =.017). Tukey’s post hoc test demonstrated that recipients implanted with the CSS group were significantly younger than the MFX group (*p* =.013), while no other pairwise differences were significant. The mean AID also differed significantly among the three electrode array groups (F(2, 145) = 33.50, *p* <.001). Tukey’s HSD post-hoc analysis showed that the MFX group had significantly greater mean AID than both the CSS group (mean difference = 81°, *p* <.001) and the ABSJ group (mean difference = 71°, *p* <.001), whereas AID did not differ significantly between the latter two electrode array groups (*p* =.627).

Inter-observer reliability was good to excellent across all measurements. The intraclass correlation coefficient (ICC, two-way random-effects model, absolute-agreement type) indicated excellent reliability for Distance A (ICC = 0.911, 95% CI 0.875–0.936, *p* <.001) and AID (ICC = 0.985, 95% CI 0.979–0.990, *p* <.001), and good-to-excellent reliability for Distance B (ICC = 0.883, 95% CI 0.783–0.930, *p* <.001).

### Group comparisons of scalar scores across angular positions

**Fig. 4:**
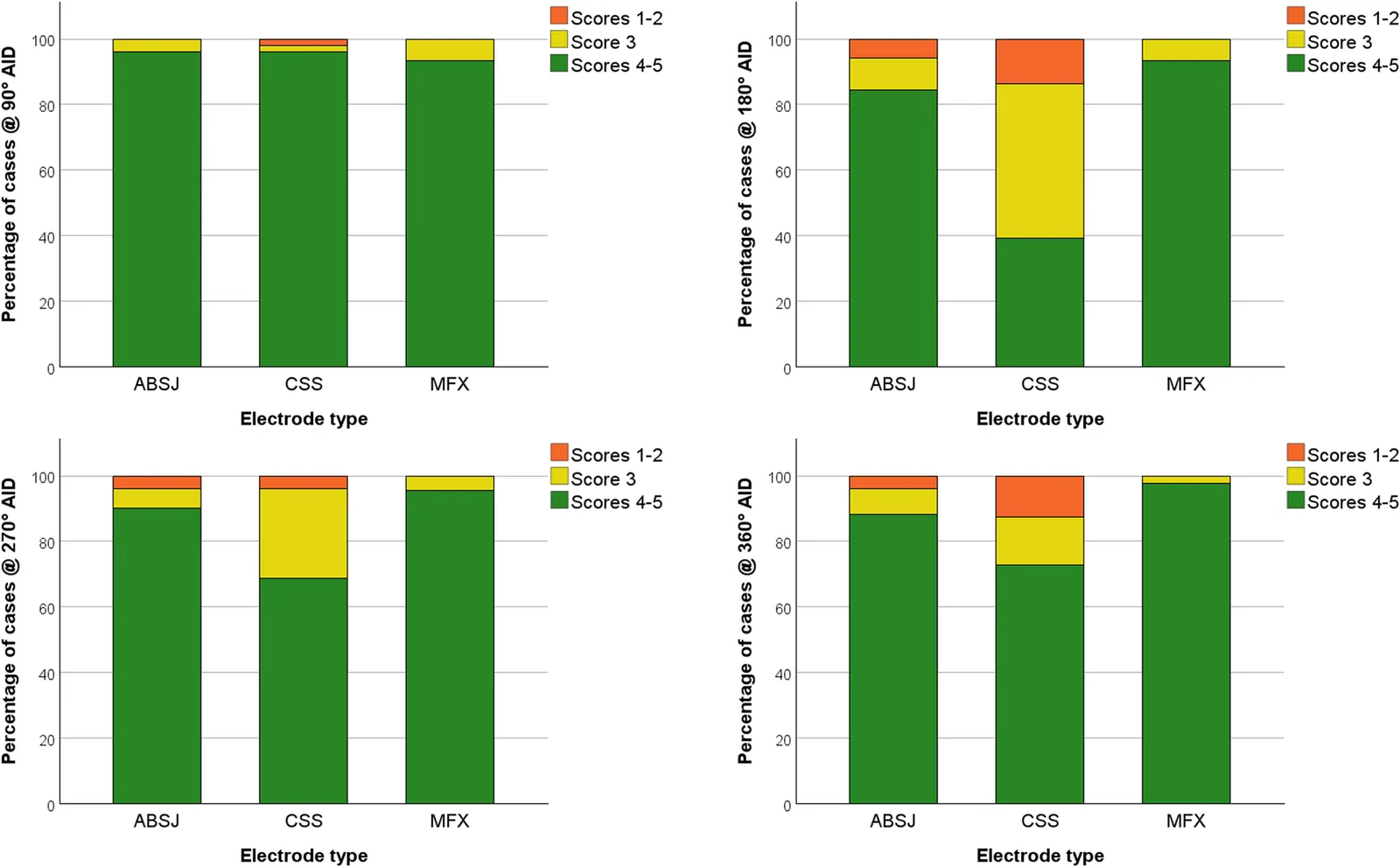
Scores represent the position of the electrode array within the cochlear scala: 1–2 = scala vestibuli (orange), 3 = central lumen (yellow), 4–5 = scala tympani (green). Data are presented as percentage of cases at each angle.

Since the scalar position scores were ordinal and summarised by median values clustered closely together across categories at each angular position, group means were not calculated. Instead, stacked bar charts are used to provide visual comparisons of score distributions across the scala chambers for each electrode array group at each angle (Fig. 4).

At all four angles (90°, 180°, 270° and 360°) there was a significant overall difference in the median scalar scores among the three electrode array groups (H(2) = 25.41, *p* <.001; H(2) = 106.06, *p* <.001; H(2) = 74.19, *p* <.001; and H(2) = 66.02, *p* <.001, respectively). Post-hoc pairwise comparisons showed that the MFX group had significantly higher scalar scores than the CSS group at every angle (all *p* <.001 after Bonferroni adjustment), and the ABSJ group at 180°, 270°, and 360° (all *p* <.001 after Bonferroni adjustment). The MFX group had a significantly higher scalar score than the ABSJ group also at 90° (*p* =.043), but the difference was not significant after Bonferroni adjustment (adjusted *p* =.129). There was no significant difference in the median scalar scores between the ABSJ group and the CSS group at any angle (all *p* >.05).

Interobserver reliability for scalar scores was moderate for individual readers (single-measure ICC = 0.42–0.58) and improved to substantial or excellent when scores were combined (average-measure ICC = 0.69–0.80). Cronbach’s α values (0.70–0.81) confirmed good internal consistency, while pairwise Cohen’s κ coefficients (0.18–0.40) indicated fair to moderate interobserver agreement across all angles.

### Group comparisons for intracochlear positions - full cohort

According to the imaging definitions used in this study, there were 47 Class A (47/51, 92.2%), no Class B, and four Class C (4/51, 7.8%) cases in the ABSJ group. There were 40 Class A (40/51, 78.4%), three Class B (3/51, 5.9%), and eight Class C (8/51 = 15.7%) cases in the CSS group. This included three cases in which the first decrease to a score of 2 or 1 was observed at 360°; in all three, the median scalar score did not increase to 4 or 5 when followed to the most apical contact, and they were therefore classified as Class C. There were no Class B or Class C cases in the MFX group (46/46, 100% Class A). In the ABSJ group, the distribution of quadrants in which STL occurred was as follows: 0–90, *n* = 0/4; 90–180, *n* = 3/4; 180–270, *n* = 0/4, 270–360, *n* = 1/4. In the CSS group, the distribution of quadrants in which STL occurred was as follows: 0–90, *n* = 1/8; 90–180, *n* = 3/8; 180–270, *n* = 1/8, 270–360, *n* = 3/8.

In the STL vs. no-STL analysis, the overall difference in STL frequency amongst the three electrode array groups was statistically significant (Fisher-Freeman-Halton exact test, *p* =.012). In the post hoc pairwise comparisons, the STL frequency did not differ significantly between the ABSJ group and the CSS group (Fisher’s exact test, *p* =.357; odds ratio 2.19, 95% CI 0.61–7.78), or between the ABSJ group and the MFX group (*p* =.119; odds ratio not estimable due to a zero cell). In contrast, the STL frequency was significantly lower in the MFX group than the CSS group (*p* =.006; odds ratio not estimable due to a zero cell), and this difference remained significant after Bonferroni adjustment for multiple comparisons (adjusted *p* =.018).

In the ST vs. not-ST analysis, the overall difference in ST retention amongst the three electrode array groups was statistically significant (Fisher-Freeman-Halton exact test, *p* <.001). In the post hoc pairwise comparisons, the ST retention frequency did not differ significantly between the ABSJ group and the CSS group (Fisher’s exact test, *p* =.091; odds ratio 0.31, 95% CI 0.09–1.05), or between the ABSJ group and the MFX group (*p* =.119; odds ratio not estimable due to a zero cell). In contrast, the ST retention frequency was significantly higher in the MFX group than the CSS group (*p* <.001; odds ratio not estimable due to a zero cell), and this difference remained significant after Bonferroni adjustment for multiple comparisons (adjusted *p* <.003).

### Group comparisons for intracochlear positions - sensitivity analysis

To verify that the differences in the group comparisons were not driven by cases in which the first decrease to a score of 1 or 2 occurred at 360°, a sensitivity analysis was performed after excluding these cases (*n* = 144). In this restricted dataset, there were 47 Class A (47/50, 94.0%), no Class B, and three Class C (3/50, 6.0%) cases in the ABSJ group. There were 40 Class A (40/50, 80.0%), three Class B (3/50, 6.0%), and five Class C (5/50 = 10.0%) cases in the CSS group. There were no Class B or Class C cases in the MFX group (46/46, 100% Class A).

In this sensitivity analysis, the overall difference in STL frequency (STL vs. no-STL) was no longer significant (Fisher-Freeman-Halton exact test, *p* =.088). However, there remained a significant overall difference amongst the three electrode array groups in ST retention frequency (*p* =.005). In the post hoc pairwise comparisons, the ST retention frequency did not differ significantly between the ABSJ group and the CSS group (Fisher’s exact test, *p* =.117; odds ratio 0.319, 95% CI 0.079–1.284), or between the ABSJ group and the MFX group (*p* =.243; odds ratio not estimable due to a zero cell). In contrast, the ST retention frequency was significantly higher in the MFX group than the CSS group (*p* =.006; odds ratio not estimable due to a zero cell), and this difference remained significant after Bonferroni adjustment for multiple comparisons (adjusted *p* =.018).

### Association between covariate parameters and intracochlear position characteristics

Binary comparisons were performed to assess whether there was any association between cochlear basal turn dimensions or AID and intracochlear position characteristics. Analyses were conducted separately for the ABSJ group and the CSS group, but not for the MFX group as the latter had no case of Class B or Class C. Distance A, Distance B, and AID were entered as continuous predictor variables in binary logistic regression models, with STL (STL vs. no-STL) and ST retention (ST vs. not-ST) as binary outcomes.

Across all analyses, none of the regression models reached statistical significance (χ² range 1.49–2.14, all *p* ≥.54). No independent associations were identified between cochlear basal turn dimensions (distance A, distance B) or AID and either STL or ST retention. All models showed satisfactory calibration (Hosmer-Lemeshow *p* =.68-.69) but minimal explanatory power (Nagelkerke R² = 0.06–0.07).

All results are summarised in Table 3.

**Table 3:** Summary of cohort characteristics, cochlear measurements, intracochlear position characteristics, and statistical comparisons among the three electrode array groups

Parameter	Advanced Bionics SlimJ (*n* = 51)	Cochlear Slim Straight (*n* = 51)	MED-EL FLEX (*n* = 46)	Overall/Notes
Implanted ear side	32 R, 19 L	22 R, 29 L	25 R, 21 L	χ² = 3.97, df = 2, *p* =.138 → ns
Distance A (mm)	9.1 ± 0.4	9.1 ± 0.5	9.2 ± 0.4	F(2,145) = 0.093, *p* =.911 → ns
Distance B (mm)	6.7 ± 0.4	6.9 ± 0.4	6.8 ± 0.4	F(2,145) = 1.650, *p* =.196 → ns
PTA4 (dB HL)				
Preoperative (*n* = 148)	100 ± 15	95 ± 15 dB	95 ± 16	F(2, 145) = 2.094, *p* =.127 → ns
Postoperative (*n* = 122)	112 ± 10	109 ± 15	109 ± 12	F(2, 118) = 0.687, *p* =.505 → ns
LFPTA3 (dB HL)				
Preoperative (*n* = 148)	88 ± 18	78 ± 22	74 ± 27	ABSJ > CSS (+ 11 dB HL, *p* =.025) ABSJ > MFX (+ 14 dB HL, *p* =.010) CSS vs. MFX *p* =.758→ ns
Postoperative (*n* = 122)	102 ± 16	96 ± 20	93 ± 20	F(2, 118) = 2.365, *p* =.098 → ns
Age at implantation (years)	53.9 ± 23.3	46.1 ± 24.9	59.5 ± 20.1	CSS < MFX (*p* =.013*)
AID (°)	436 ± 46	426 ± 53	507 ± 58	MFX > CSS (+ 81°, *p* <.001*) MFX > ABSJ (+ 71°, *p* <.001*) ABSJ vs. CSS *p* =.627→ ns
Inter-observer reliability (ICC, 95% CI)	Distance A 0.911 (0.875–0.936); Distance B 0.883 (0.783–0.930); AID 0.985 (0.979–0.990); all *p* <.001* → good to excellent reliability			
Median scalar scores (group comparison at each angle)	MFX > CSS (all adjusted *p* <.001*) and > AB at 180°, 270°, 360° (adjusted *p* <.001*) ABSJ vs. CSS → ns (all *p* >.05)			
Inter-observer reliability and agreement (scalar scores)	Single-measure ICC 0.42–0.58 (moderate); Average-measure ICC 0.69–0.80 (substantial to excellent); Cronbach’s α 0.70–0.81 (good); Cohen’s κ 0.18–0.40 (fair to moderate)			
Intracochlear position characteristics (full *n* = 148)	Class A 47 (92.2%) Class B 0 Class C 4 (7.8%)	Class A 40 (78.4%) Class B 3 (5.9%) Class C 8 (15.7%)	Class A 46 (100%) Class B 0 Class C 0	STL vs. no STL *p* =.012*; ST vs. not ST *p* <.001*
STL vs. no-STL (full *n* = 148)	4 STL (7.8%) 47 no STL (92.2%)	8 STL (15.7%) 43 no STL (84.3%)	0 STL (0%) 46 no STL (100%)	ABSJ vs. CSS *p* =.357 → ns; ABSJ vs. MFX *p* =.119 → ns; CSS vs. MFX *p* =.006* (adjusted *p* =.018*)
ST vs. not-ST (full *n* = 148)	ST 47 (92.2%) not ST 4 (7.8%)	ST 40 (78.4%) not ST 11 (21.6%)	ST 46 (100%) not ST 0 (0%)	ABSJ vs. CSS *p* =.091 → ns; ABSJ vs. MFX *p* =.119 → ns; CSS vs. MFX *p* <.001* (adjusted *p* <.003*)
Intracochlear position characteristics (sensitivity *n* = 144)	Class A 47 (94.0%) Class B 0 Class C 3 (6.0%)	Class A 40 (80.0%) Class B 3 (6.0%) Class C 5 (10.0%)	Class A 46 (100%) Class B 0 Class C 0	STL vs. no STL *p* =.088 → ns; ST vs. not ST *p* =.005*
STL vs. no STL (sensitivity *n* = 144)	3 STL (6.0%) 47 no STL (94.0%)	5 STL (10.0%) 45 no STL (90.0%)	0 STL (0%) 46 no STL (100%)	Overall *p* =.088 → ns
ST vs. not ST (sensitivity *n* = 144)	ST 47 (94.0%) not ST 3 (6.0%)	ST 40 (80.0%) not ST 10 (20.0%)	ST 46 (100%) not ST 0 (0%)	ABSJ vs. CSS *p* =.117 → ns; ABSJ vs. MFX *p* =.243 → ns; CSS vs. MFX *p* =.006* (adjusted *p* =.018*)
Binary logistic regression (Cochlear and AB only)	χ² = 1.49–2.14, *p* ≥.54; Nagelkerke R² = 0.06–0.07 (low explanatory power); Hosmer–Lemeshow *p* =.68-0.69 → good fit; no significant predictors (distance A, distance B, AID) for STL or ST retention			

R, right ear; L, left ear; p, probability value (two-sided); ns, not significant; ±, mean ± standard deviation; CSS, Cochlear Straight Slim; MFX, MED-EL FLEX; ABSJ, Advanced Bionics SlimJ; PTA4, pure-tone thresholds averaged over 0.5, 1, 2, and 4 kHz; LFPTA3, low-frequency pure-tone thresholds averaged over 0.25, 0.5 and 1 kHz; AID, angular insertion depth; ICC, intraclass correlation coefficient; CI, confidence interval; Class A, all scalar scores ≥ 3; Class B, scalar score decreases to ≤ 2 before increasing to ≥ 4; Class C, scalar score decreases to ≤ 2 and does not increase to > 3; STL, scalar translocation; ST, scala tympani

## Discussion

This retrospective study evaluated postoperative cone-beam CT scans of 148 morphologically normal cochleae implanted with three different types of lateral wall (LW) electrodes: Advanced Bionics SlimJ (ABSJ), Cochlear Slim Straight (CSS), and MED-EL FLEX (MFX) arrays. The aim of the study was to determine differences in intracochlear position characteristics and scalar translocation (STL) rates within the cochlear basal turn. Scalar location was assessed using a five-point linear grading system (scores 1 and 2, scala vestibuli, SV; scores 4 and 5, scala tympani, ST) at 90°, 180°, 270° and 360°, with positions categorised into three classes: Class A (ST retention), Class B (basilar-membrane elevation or rupture), and Class C (STL). Quantitative measurements of cochlear basal-turn dimensions and angular insertion depth (AID) assessed potential anatomical confounders. Across the full cohort, STL occurred in 12 of 148 cases (8.1%), while complete ST retention was observed in 133 of 148 cases (89.9%). The overall frequency of STL and ST retention differed significantly among the three LW electrode types, with STL observed most often in the CSS and not at all in the MFX group. Median scalar scores were significantly higher for the MFX group compared with the CSS group at every angle (all Bonferroni-adjusted *p* <.001) and compared with the ABSJ group at 180°, 270°, and 360° (all *p* <.001). No associations were identified between cochlear dimensions or AID and scalar position. Inter-observer reliability was excellent for quantitative measurements and fair to substantial for the ordinal scalar scores.

The overall STL rate observed in the present study (8.1%) aligns with meta-analyses of lateral wall electrodes by Jwair et al. [4] and Dhanasingh et al. [27], which reported rates of 7% and 6.7%, respectively. A systematic review by van de Heyning et al. [28] reported a slightly higher STL rate of 11% for all types of LW electrodes. However, among individual studies, reported STL rates have ranged from 0% to 20%, a relatively wide variation in findings that is not unexpected given the differences in the specific types of lateral wall electrodes examined, the small sample sizes in many series, the surgical approach to insertion (round window (RW) versus cochleostomy), and the varying methodologies used to determine scalar translocation. In the present study, potential covariates were minimised by restricting the inclusion criteria to morphologically normal cochleae on preoperative MRI, full insertions via the RW, and postoperative imaging with CBCT, which has been shown to provide higher spatial resolution, reduced metal artifacts from electrode contacts and a higher diagnostic accuracy than CT for intracochlear localisation of the CI electrode array [29, 30].

A number of different definitions and methods have been reported for the determination of STL in vivo. One readily accessible approach involves reformatting postoperative CT or CBCT images and diagnosing STL when the electrode array crosses from posterior (SV) to anterior (ST) [18, 22]. The present study adopted this method and, to minimise potential error from interobserver variability, three observers independently applied a systematic approach to scalar scoring, with the median score used for analysis. The proposed imaging classification enabled translation of intermediate or indeterminate electrode positions into Eshraghi grades 1 and 2, which in turn facilitated the distinction between STL and ST retention, since absence of definite STL does not necessarily indicate ST retention. Furthermore, this classification provided greater granularity in describing the scalar position of each type of LW electrode array at different angular depths within the cochlea. However, accurate determination of electrode location within the cochlear lumen becomes increasingly challenging beyond the basal turn on axial imaging [31, 32]. The analysis in the present study was therefore confined to the basal turn, where scalar position could be assessed with greater reliability.

Another approach utilises software-based processing of postoperative CT or CBCT images, using cochlear and electrode models generated from micro-CT of temporal bones [33, 34], imaging fusion [35, 36], or atlas-based segmentation [37, 38]. Such methods have the distinct advantage of enabling the assessment of scalar position beyond the basal turn. However, it should be recognised that the diagnostic accuracy of electrode position assessment depends on the generalisability of the model, the precision of the registration, and the quality of imaging [39, 40].

In the only other study with sufficient sample size that examined different types of LW electrode arrays directly, Morrel et al. [41] applied a statistical shape model method to postoperative CT or CBCT to determine STL in 177 ears and found an overall 22% STL rate, with no significant difference among different types of LW electrode arrays. The authors reported a median translocation depth of 381°, with most translocations occurring around 400° and a smaller subset around 200°, mainly involving the Cochlear CI422/522 arrays. They also found a significant correlation between angular insertion depth (AID) and translocation rate. Comparison of the findings of Morrel et al. with those of the present study is challenging for several reasons. First, Morrel et al. used a statistical shape model approach and assessed electrode arrays along their entire trajectories, extending to the apical contacts, whereas the present study was limited to the basal turn. Second, while the present study included a relatively balanced distribution of lateral wall electrode types (ABSJ *n* = 51, CSS *n* = 51, MFX *n* = 46), the cohort of Morrel et al. was heavily weighted towards MED-EL electrodes (92/177; 52%) and contained only 11 ABSJ arrays (6%). Given the higher mean AID reported, it is probable that an increasing proportion of MED-EL electrodes contributed to the deeper insertions and associated STL observed. Third, cohort and procedural details were not available for comparison, including case characteristics, inclusion and exclusion criteria, insertion technique (round window versus cochleostomy), imaging modality (CT or CBCT for STL cases), and the distribution and locations of STLs among different LW electrode types.

In the current study, a possible pattern of STLs was observed in both the ABSJ and CSS groups, with a cluster in the 90–180° quadrant (ascending segment) and a smaller cluster in the 270–360° quadrant (descending segment), although some STLs also occurred in other regions. Given the small numbers and low overall STL rates, no meaningful statistical analysis was possible. A wide range of “typical” STL locations, expressed as angular depth, has been reported for LW electrodes across studies using different methodologies, ranging from approximately 150° to well beyond 360°, with no consistent pattern attributable to electrode array type [24, 41–45]. No correlation was found in the present study between cochlear basal turn dimensions and STL rate, in agreement with Ketterer et al. [45]. This contrasts with the findings of the study on a pre-curved electrode array by Pai et al. [17], which reported a strong correlation between distance A and STL.

The authors acknowledge that there are some limitations to the present study. First, as already discussed, the present study only assessed the basal turn of the cochlea. Whilst the findings suggest that MFX electrode arrays are significantly more likely to remain in the ST compartment than the CSS electrode arrays, it must be remembered that this only applies to the basal turn, and it is entirely plausible that the MFX and ABSJ electrode arrays begin translocating beyond 360°, such that there is no overall difference among the different LW electrode array types. An assessment tool readily applicable in clinical practice, without concerns about errors introduced during post-processing, would be highly valuable. Second, as the point spread function of electrode contacts is easier to define with the MFX and ABSJ electrode arrays which have fewer electrode contacts, this may have an unavoidable impact on the assessment of the scalar position (observation bias). Third, the retrospective design of the study innately introduces selection bias.

## Conclusion

The findings from the present study demonstrate that, even among LW arrays, there is a significant difference in scalar position and STL rates within the cochlear basal turn, with the MFX electrode arrays showing the most stable ST placement. Differences in scalar behaviour do not appear to be related to basal turn dimensions and may instead reflect the inherent physical properties of the arrays. Although confined to the basal turn, the study outlines a systematic methodology and imaging classification for determining the scalar position of cochlear implant electrode arrays. A practical approach that can be readily applied to clinical axial imaging for broader assessment beyond 360° will be highly valuable.

## Electronic Supplementary Material

Below is the link to the electronic supplementary material.

Supplementary Material 1 (DOCX 17.7 KB)

## Data Availability

Non-identifiable data are available from the corresponding author upon reasonable request and subject to appropriate review.
