# Supplementary material for: Scalar position characteristics and translocation rates of different lateral wall electrodes within the cochlear basal turn: a cone beam computed tomography study
Source: Eur Arch Otorhinolaryngol. 2026 Aug 7;283(10):6379–90. doi: 10.1007/s00405-026-10431-5 (PMC13615049; doi:10.1007/s00405-026-10431-5)
Supplement: Supplementary file 1 [file 405_2026_10431_MOESM1_ESM.docx]

Supplementary Table 1. Distribution of excluded ears by electrode array type and reason for exclusion.
Array type
Active contacts lateral to RW 
Explant/re-implantation 
Cochleostomy 
Compromised basal turn patency 
Poor image quality 
IP-II 
Insufficient clinical data
Total excluded
ABSJ
4
3
3
7
6
2
2
27
CSS
3
4
2
0
0
2
0
11
MFX
1
1
2
0
0
0
0
4
Total
8
8
7
7
6
4
2
42

ABSJ, Advanced Bionics SlimJ; CSS, Cochlear Slim Straight; MFX, MED-EL FLEX; RW, round window; IP-II, Incomplete partition type 2.
